# Supplementary material for: Behavioral and social predictors of suicidal ideation and attempts among adolescents and young adults
Source: PLOS Ment Health. 2025 Jan 3;2(1):e0000221. doi: 10.1371/journal.pmen.0000221 (PMC12798384; doi:10.1371/journal.pmen.0000221)
Supplement: Attachment — Submitted filename: Response to Reviewers.docx [file pmen.0000221.s001.docx]

**Editor’s comments**
Thank you for submitting your manuscript to this journal. Kindly address the following comments

**Response: Thank you for all the constructive feedback. We have made all the changes as per your suggestions.**

Abstract:
Conclude based on your findings
**Response: We have revised the conclusion and deleted the far-fetched implications.**

Background:
Well-focused, provided context but requires grammatical editing
**Response: We have proofread the manuscript and corrected grammatical errors.**

Methods:
• Specify what age range was considered in this study

**Response: YRBSS (our data source) does not specify exact age, but it does specify school grades of the respondents, i.e. grade 9 to 12. Typically, in the US student in these grades would typically be between the age range of 13 to 19 years. In the manuscript we have mentioned the school grades instead of age estimates.**

• The use of only item to determine suicidal idea may overestimate it and may be considered a potential limitation

**Response: We agree. We have acknowledged the same as a limitation of our study.**

***“****[…] the use of a single item to determine suicidal ideation may overestimate its prevalence, potentially affecting the accuracy and reliability of our predictions […]”*

• The grammatical errors persisted in the method section, kindly carry out grammar editing
**Response: We have rectified them.**

Results:
Well-presented
**Response: Thank you .**

Discussion:
How comparable is your findings to the global literature
**Response: We already compared our model with the literature using table 4. However, to further make it easy to read, we have briefly summarized this comparisons in a paragraph referring back to the table.**

*“Unlike many existing studies (Table 4), which rely heavily on clinical or latent constructs such as severe depression, anxiety, previous suicide attempts, antisocial score, or perceived warmth, our approach uses data that are more accessible and observable by public, like teachers, parents, or friends. This makes our model not just innovative but also more practical for real-world prevention efforts. Besides, the essence of using ML models in suicide prevention is to uncover hidden patterns that aren't immediately obvious to humans. If we only used variables that clearly indicate a high risk of reattempt, such as a history of suicide attempts or severe depression, we wouldn't be fully utilizing the power of ML. Such information, while crucial, might not help in early prevention because it's already an established risk factor. Our study reveals new insights that can guide prevention strategies. For example, our model, using the YRBSS, identified predictors like being electronically bullied, using an electronic vapor product, experiencing sexual violence, and misusing prescription pain medicine, achieving an AUROC of 0.82 for suicidal ideation and 0.83 for suicide attempts, with an accuracy of 0.74 for both. In contrast, other studies, such as those using the Health Research Enterprise from French universities [39] or the Minnesota Multiphasic Personality Inventory-2 Restructured Form [40], focus on predictors like trait anxiety, depression, and family problems, achieving similar or slightly higher AUROC scores but often relying on less observable factors. Therefore, our model's emphasis on accessible data offers a significant advantage in practical applications for early intervention and prevention efforts.”*

Limitation:
List potential limitations of this study

**Response: We have added a limitation section.**

***“****Our study has few limitations that should be considered. First, the use of a single item to determine suicidal ideation may overestimate its prevalence, potentially affecting the accuracy and reliability of our predictions. Second, while the YRBSS provides a comprehensive dataset, it relies on self-reported data, which may introduce biases such as underreporting or misreporting of sensitive behaviors and experiences. Third, our model was developed and validated using data from students in grades 9 to 12 in the United States, which may limit the generalizability of our findings to other populations or age groups. Additionally, the cross-sectional nature of the YRBSS data prevents us from making causal inferences about the specific directional causal relationships between the predictors and suicidal behaviors. Finally, our study focuses on social and behavioral factors, which, while important, do not encompass all potential risk factors for suicidal ideation and attempts, such as genetic, biological, or environmental influences. Future research should consider integrating these additional dimensions to provide a more holistic understanding of the predictors of suicidal behaviors.”*

Conclusion:
Stick to your findings
**Response: We have revised the conclusion.**

***“****Our study provides critical insights into the predictors of suicidal ideation and attempts among youth, highlighting the significant impact of cyberbullying, drug use, sexual violence, misuse of prescription medicine, and others. By understanding the significant impact of these factors, families are better equipped to recognize warning signs and intervene effectively, and it highlights the need for families to create supportive home environments that promote mental well-being and resilience. Additionally, this research advocates for the urgent need for support systems that address the early signs of suicidal behaviors.”*

Reviewers' comments: **None available**
