# Supplementary material for: Behavioral and social predictors of suicidal ideation and attempts among adolescents and young adults
Source: PLOS Ment Health. 2025 Jan 3;2(1):e0000221. doi: 10.1371/journal.pmen.0000221 (PMC12798384; doi:10.1371/journal.pmen.0000221)
Supplement: Attachment — Submitted filename: Response to Reviewers_2.docx [file pmen.0000221.s002.docx]

**Reviewer #1**

This is a well written manuscript. Some the references do not have neither page numbers nor doi., and some web page cited do not have URL. Please go through the reference and make corrections.

**Response: Thank you for the constructive feedback. We have reviewed and corrected all the references, adding page numbers, DOIs, and URLs where necessary.**

**Reviewer #2:**

Abstract:

• Line 2 should read “The study aimed to determine the social and behavioral predictors……………”

**Response: We have revised line 2 as suggested.**

• Write the abbreviations in full before subsequently abbreviating e.g., PPV, AUROC etc.

**Response: We have expanded all abbreviations to their full forms in the abstract.**

“The model identified cyberbullying, drug use, and sexual violence, difficulty in 42 concentrating, early initiation of alcohol or marijuana, as some of the most important predictors”

• Predictors of what?

**Response: We have clarified the sentence by adding "predictors of suicidal ideation and attempts."**

• Kindly insert the test statistics e.g., the odd ratio and its 95% confidence interval

**Response: We have added the odds ratio and its 95% confidence interval to the manuscript.**

• Keywords: Add “social” and “behavioral predictors”

**Response: We have included “social” and “behavioral predictors” in the keywords.**

Background

• Rephrase the objective in the last paragraph as suggested in the abstract

**Response: We have rephrased the objective to “social and behavioral predictors of suicidal ideation and attempts.”**

Methods

• Is appropriate

**Response: Thank you.**

Results

• Add the 95% confidence interval for the prevalence of suicidal ideation and attempt

**Response: We have added the prevalence of suicidal ideation and attempts.**
