# Supplementary material for: Behavioral and social predictors of suicidal ideation and attempts among adolescents and young adults
Source: PLOS Ment Health. 2025 Jan 3;2(1):e0000221. doi: 10.1371/journal.pmen.0000221 (PMC12798384; doi:10.1371/journal.pmen.0000221)
Supplement: Attachment — Submitted filename: Response to Reviewers_3.docx [file pmen.0000221.s003.docx]

Reviewer #3: The authors identify the behavioural and social predictors of suicidal ideation and atempts among adolescents and young adults in the US. This is an important piece that warrants publication in the journal.

**Response: Thank you so much.**

I have only two comments:

1. While poetic, the opening sentences in the study can be quite triggering for readers, especially for those with lived experience of suicide beravement. I would suggest changing it.

**Response:** I totally agree. I have changed the opening sentences.

*“The question of 'why' often lingers after the loss of a loved one to suicide, accompanied by reflections on moments missed and support unoffered. This reality underscores the complex and often unseen nature of mental health struggles.”*

2. While the study uses a novel method of analysis, the relationships between the predictors and SI and SA are well established in the literature. Thus, I would urge the authors to temper their language of the novelty of the findings. This would serve well, given that until today, even machine learning methods struggle to predict who is likely to attempt suicide - leading to the widely accepted conclusion that utilising factors relating to suicide to prevent suicide are challenging.

**Response:** To address this point. I made changes in the introduction last paragraph, discussion first paragraph, and rewrote the conclusion.

Introduction:

*“Our study aims to identify and leverage social and behavioral predictors of suicidal ideation and attempts among adolescents and young adults. Note that the objective is not to introduce a new predictive model as a replacement of clinical assessment but to assess if social and behavioral predictors that are observable by friends, family, or individuals themselves, can help predict suicidal ideation or attempt that a layperson can use as a preliminary tool at home.”*

Discussion:

“While we acknowledge that the relationships between the predictors of SI and SA are well established in the literature, our study brings a fresh perspective to predicting thoughts of suicide and suicide attempts by focusing on observable, everyday behaviors and experiences of young people, such as experiences of bullying, drug use (including alcohol, cigarettes, marijuana, and vapor products), experiences of sexual and physical violence, and difficulties with concentration (like remembering things or making decisions).”

Conclusion:

*“Our study provides critical insights into the predictors of suicidal ideation and attempts among youth, highlighting the significant impact of factors such as cyberbullying, drug use, sexual violence, and misuse of prescription medications. By understanding these influences, families are better equipped to recognize warning signs and intervene effectively, underscoring the need to create supportive home environments that promote mental well-being and resilience. Additionally, this research advocates for the urgent development of support systems that address the early signs of suicidal behaviors. Aligning with the widely accepted conclusion, our study also surfaces the fact that predicting with certainty who is likely to attempt suicide remains a challenge.”*
